# Supplementary material for: Prediction of anemia and estimation of hemoglobin concentration using a smartphone camera
Source: PLoS One. 2021 Jul 14;16(7):e0253495. doi: 10.1371/journal.pone.0253495 (PMC8279386; doi:10.1371/journal.pone.0253495)
Supplement: S1 Table — 26 image-based parameters extracted from the ROI. Each image was processed to derive this set of parameters. (DOCX) [file pone.0253495.s001.docx]

**S1 Table. Image-Based Parameters**

| Symbol | Imaged-based parameter |
| --- | --- |
| BRIGHT | Average value of gray scale image |
| R0 | Average value of red component of all pixels |
| R1 | Average value of red component of pixels between 2nd and 12th percentiles |
| R2 | Average value of red component of pixels between 50th and 52nd percentiles |
| R3 | Average value of red component of pixels between 88th and 98th percentiles |
| G0 | Average value of green component of all pixels |
| G1 | Average value of green component of pixels between 2nd and 12th percentiles |
| G2 | Average value of green component of pixels between 50th and 52nd percentiles |
| G3 | Average value of green component of pixels between 88th and 98th percentiles |
| B0 | Average value of blue component of all pixels |
| B1 | Average value of blue component of pixels between 2nd and 12th percentiles |
| B2 | Average value of blue component of pixels between 50th and 52nd percentiles |
| B3 | Average value of blue component of pixels between 88th and 98th percentiles |
| RPVM | Average value of red component of pixels between 40th and 60th percentiles |
| GPVM | Average value of green component of pixels between 40th and 60th percentiles |
| BPVM | Average value of blue component of pixels between 40th and 60th percentiles |
| ENROPY | Entropy of grayscale image |
| HHR | High hue ratio |
| H | Average value of hue from HSV colormap |
| L | Average value of lightness from LAB colormap |
| FLASS | Did camera flash fire for this image? |
| BE | Camera metadata: Baseline Exposure |
| ASN1 | Camera metadata: AsShotNeutral1 |
| ASN2 | Camera metadata: AsShotNeutral2 |
| BV | Camera metadata: BrightnessValue |
| Hb | Hemoglobin measured. Not used for predictions rather reference for testing. |

26 image-based parameters extracted from the ROI. Each image was processed to derive this set of parameters.
