# Supplementary figures and images for: Prediction of anemia and estimation of hemoglobin concentration using a smartphone camera
Source: PLoS One. 2021 Jul 14;16(7):e0253495. doi: 10.1371/journal.pone.0253495 (PMC8279386; doi:10.1371/journal.pone.0253495)

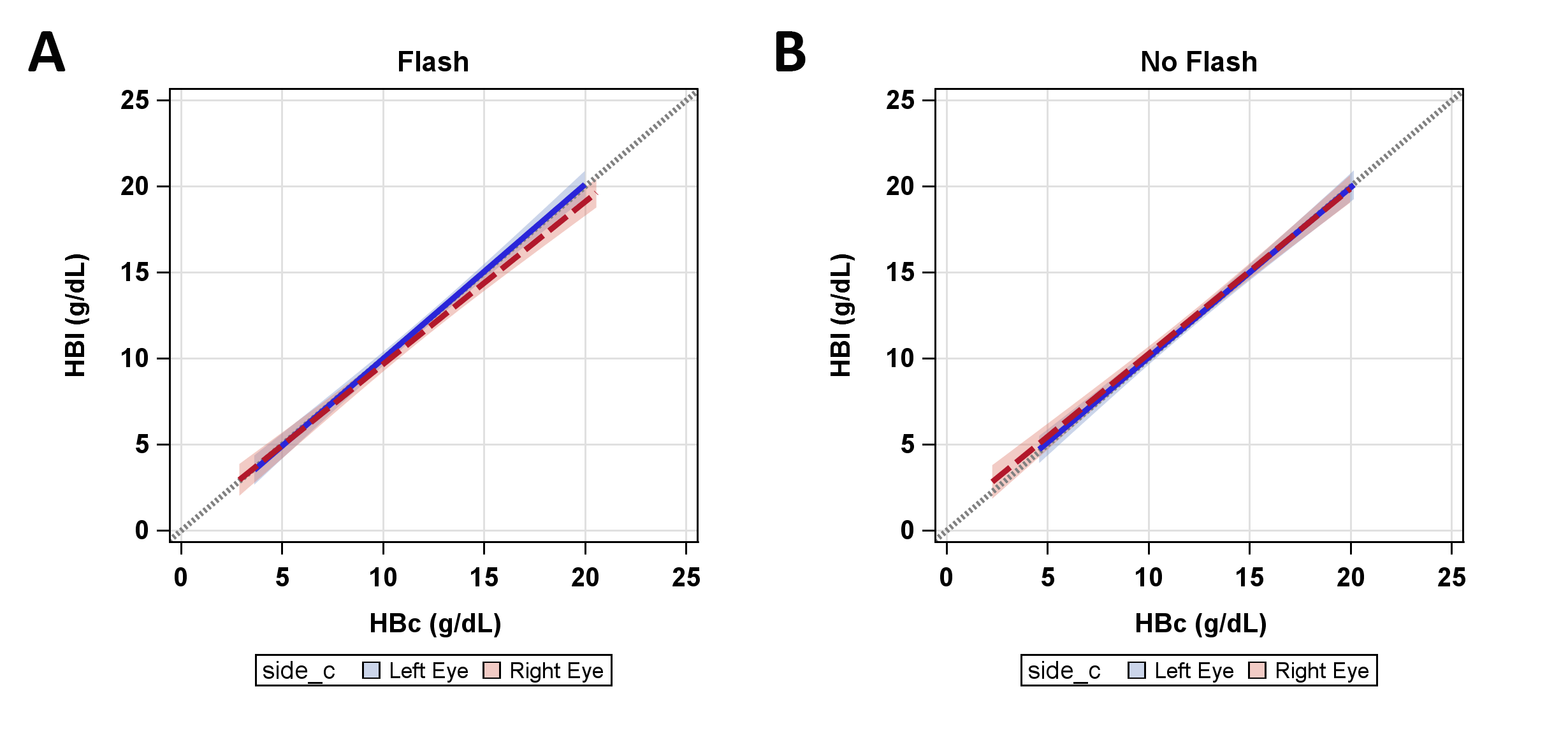

Supplement: S1 Fig — The graph on the left shows the correlation when flash was used, and the graph on the right shows the correlation without flash. The x-axis depicts HBc in g/dL, the y-axis HBl. The red line corresponds to data from the right eye and the blue line from the left. The shaded areas depict the 95% confidence intervals. The gray dotted line is the identity line (perfect agreement between HBl and HBc). (TIF) [file pone.0253495.s002.tif]

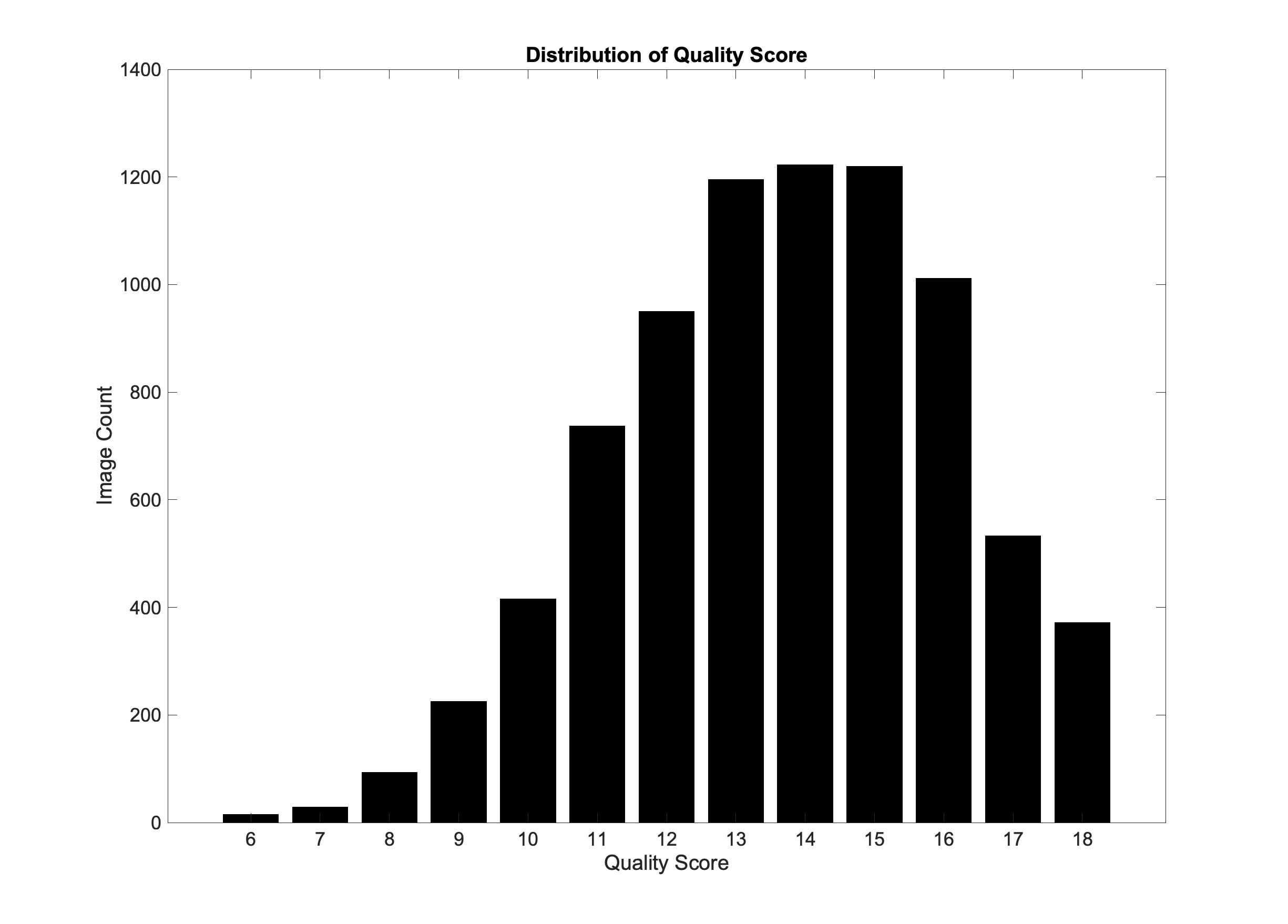

Supplement: S2 Fig — Higher scores correspond to better image quality comprised of the sum of scores for focus, extent of conjunctival exposure, and lighting. Each domain was scored on a 3-point Likert scale and summed across three observers; the maximal score was 18. (TIF) [file pone.0253495.s003.tif]
