# Supplementary material for: Prediction of anemia and estimation of hemoglobin concentration using a smartphone camera
Source: PLoS One. 2021 Jul 14;16(7):e0253495. doi: 10.1371/journal.pone.0253495 (PMC8279386; doi:10.1371/journal.pone.0253495)
Supplement: Attachment — Submitted filename: REVIEW.docx [file pone.0253495.s004.docx]

The paper was written well scientifically good . Lot of efforts has been done.

1. Enumerate the reason for selecting palpebral conjunctiva pallor for assessment of anaemia.
2. Two Different type of image formatS JPEG and PNG are used.What is the reason behind that?.
3. How the linear model is better than other model for modeling error	` ?
4. What is sandwich estimation
5. does the size of the dataset affect the Sensitivity, Specificity and prevalence of the disease?
6. Does population with higher prevalence, affect the positive and negative predictive value.
7. specify the reason for choosing HSI colour space for anaemia estimation.
