# Supplementary material for: Prediction of anemia and estimation of hemoglobin concentration using a smartphone camera
Source: PLoS One. 2021 Jul 14;16(7):e0253495. doi: 10.1371/journal.pone.0253495 (PMC8279386; doi:10.1371/journal.pone.0253495)
Supplement: Attachment — Submitted filename: Response to Reviewers 24.05.2021.doc [file pone.0253495.s005.doc]

May 20, 2021

Dear Drs. McBride, Turner, Misra and the *PLOS Medicine* Editorial Board,

We are enclosing the ***revision*** to manuscript entitled “Prediction of Anemia and Estimation of Hemoglobin Concentration Using a Smartphone Camera” PONE-D-21-07161, for evaluation and potential publication as a research article in your Journal. Anemia is widely prevalent globally and has a direct impact on morbidity and mortality. This paper was initially submitted to PLOS-Medicine and then re-directed to PLOS-ONE. Our manuscript describes the use of an easily accessible device, the smartphone, to capture and process images of the palpebra conjunctiva to predict hemoglobin concentration. There are prior publications in this area by the authors, and others who report similar (or better) hemoglobin prediction accuracy. However, we have observed that there is a diminution of accuracy in the normal two-step derivation and validation process of algorithm development that other publications in this area do not report. In addition, the size of our study population is the largest reported to date involving imaging of the conjunctiva for hemoglobin estimation. We believe that the information covered in this manuscript is highly pertinent to the medical community as it has real-world clinical implications as a screening tool for anemia. This is also more pertinent as a result of social distancing measures in pandemic management. PLOS-ONE has a high impact factor in the medical field with open access and therefore accessible to clinicians and the public, making it a suitable journal for dissemination of our work. The corresponding author of this manuscript is Gregory Jay, MD-PhD with contribution of the authors mentioned in the order below:

1. Selim Suner, MD, MS
2. James Rayner, MD, MS
3. Ibrahim U. Ozturan, MD
4. Geoffrey Hogan, MD
5. Caroline P. Meehan, MD
6. Alison B. Chambers, PhD
7. Janette Baird, PhD
8. Gregory D. Jay, MD PhD

With the submission of this revised manuscript, we would like to undertake that:

- The submitted work is an original manuscript;
- The author(s) received no specific funding for this work;
- There is no prior or duplicate submission or publication elsewhere of any part of the work including details of any presentation of the study as an abstract at a professional meeting;
- All authors of this research paper have directly participated in the planning, execution, or analysis of this study;
- The contents of this manuscript have not been copyrighted or published previously;
- The contents of this manuscript are not under consideration for publication elsewhere;
- The contents of this manuscript will not be copyrighted, submitted, or published elsewhere, while acceptance by PLOS-ONE is under consideration;
- There are no directly related manuscripts or abstracts, published or unpublished, by any authors of this paper
- I have read the journal's policy and the authors of this manuscript have the following competing interests: GD Jay and S. Suner authored US Patent #7,711,403. This does not alter our adherence to PLOS ONE policies on sharing data and materials.
- There are no new competing interests since our original submission.

Editorial comments:

1- We have ensured that our manuscript meets PLOS ONE's style requirements, including those for file naming.
2- We have included our tables as part of our main manuscript and removed the individual files.
3- We have provided additional details regarding participant consent. In the ethics statement in the Methods and online submission information, we have specified the type of consent obtained We have amended these statements in the Methods section of the manuscript, added the same text to the “Ethics Statement” field of the submission form (via “Edit Submission”).
4- We confirm that our competing interest (GD Jay and S. Suner authored US Patent #7,711,403) does not alter our adherence to all PLOS ONE policies on sharing data and materials. We also confirm that there are no new competing interests since our original submission.
5- We have altered figure 1 to remove any image that include identifying, or potentially identifying, information of the individual patient. (Our consent process included specific permission to acquire and use patient images.). Since we are not able to contact this specific patient to obtain specific consent to include his picture, we have removed panel “A” from the figure which included identifying or potentially identifying features of his face. The remaining 2 images of this conjunctiva do not contain identifying or potentially identifying features. If this is still not acceptable, we can replace these images with images of one of the authors eyes with the required consent forms; however, we are confident that the image in its revised form does not include and identifying or potentially identifying features.

Author Comments to the Reviewer:

Reviewer 1

1- We used a convenience sample to recruit patients. This was due to availability of the research staff. We limited data collection to a single staff member to achieve consistency in image collection. The volume of patients coming to our ED during the study period was approximately 100,000 patients. About 70,000 of these patients would have had a CBC test ordered. Our cohort is a small fraction of available patients. The staff member collecting the data decided which patients to screen. The decision involved the hemoglobin value, if available to ensure a wide range of hemoglobin values in our cohort as well as other factors such as if the patient was able to consent, if their care would be affected by enrolling them to the study (if the patient was undergoing specific procedures for instance), etc. The sample was chosen to include a wide range of hemoglobin values including values at the low and high extremes as well those in the normal range to best train our prediction models. When available hemoglobin values were available in the patients’ electronic medical record which the research staff could access. [Edits have been made to the Methods]
2- The underlying cause of anemia was not taken into consideration when recruiting patients, nor was this variable recorded in the research data collection form. Patients likely had a mix of etiologies of their anemia iron deficiency, vitamin B12 deficiency, renal failure, blood loss etc.). We do not believe that the different causes of anemia have a significant effect of our prediction tool. [Edits have been made to the Methods]
3- We agree that an acceptable accuracy for diagnostic point of care devices measuring hemoglobin is in the range of +/- 1.0g/dL. The reported limits of agreement in our data exceeds this by a significant amount. However, our analyses suggest that the presented data have sufficient sensitivity and specificity to utilize this device as a screening tool. In its current form the device could be used for screening both inside the hospital and in other settings (home testing, doctor’s offices, emergency medical services, etc.) with any abnormal result requiring a confirmatory blood test. We have also shown that image quality has a significant impact on accuracy of predicted results. Further improvements in our imaging technique and future planned built-in corrections based on consistent biases in prediction curves could bring the limits of agreement down to an acceptable level for a true diagnostic tool. [Edits have been made to the Discussion]

Reviewer 2:

We appreciate the suggestion to compare the conjunctiva output with other anatomical locations.

1- The palpebral conjunctiva was selected for assessment of anemia because of the unique features of this location: A) It is easily accessible for photographic sampling, B) There are no competing chromophores between the blood vessels and the conjunctival surface to affect color, C) The distance between the tissue surface and blood vessels is very narrow and D) Environmental factors such as temperature does not significant affect the blood flow to this location. [Edits have been made to the discussion]
2- The data analysis was conducted using RAW image data files and not JPEG images. In a portion of early data acquisition JPEG images were also acquired in addition to RAW image files, but these data were not used in the analysis.
3- Many different predictive models can be utilized in similar analyses. We have explored the use of other correlative models but settled on a linear model. [Edits have been made to the Discussion]
4- What is sandwich estimation: We have modified the text in the Methods to clarify the methodology: “All models were analyzed using proc glimmix unless otherwise stated. Nesting for patient and reviewer repeated measures was accounted for by modeling random effects with the residual statement (gee). **Methods to correct for the dependence among clustered data** were used to adjust for any model misspecification.  Familywise error rate (alpha) was maintained at 0.05 using the Holm adjustment for multiple comparisons where appropriate (adjusted p-values are reported). All statistical analyses were performed using SAS version 9.4 (The SAS Institute; Cary, NC).”
5- The population size does not significantly affect the sensitivity and specificity in our large cohort of patients. We have intentionally recruited patients with a wide range of hemoglobin values assuring a baseline prevalence of anemia in our cohort.
6- We have intentionally recruited patients with a wide range of hemoglobin values ensuring a baseline prevalence of anemia as well as high hemoglobin values.
7- HSV (also called HSL and HSI) are color spaces which mimic human vision closer than RGB (red, green, blue). Colors perceived to be similar by humans are close together in the color space. By using different color spaces specific image information can be more easily extracted. HSV is useful as it separated color hue from intensity and saturation allowing appreciation of color changes independent of lighting conditions. [Edits have been made to the Results and Discussion sections]

We sincerely appreciate your consideration and hope that this revision meets your criteria for publication.

Gregory D. Jay MD-PhD.

Brown University/Department of Emergency Medicine

1 Hoppin Street, Suite 106

Providence, RI 02903
